# Analysis of RapidArc optimization strategies using objective function values and dose‐volume histograms

**DOI:** 10.1120/jacmp.v11i1.3114

**Published:** 2009-12-03

**Authors:** Mike Oliver, Isabelle Gagne, Carmen Popescu, Will Ansbacher, Wayne A. Beckham

**Affiliations:** ^1^ Department of Medical Physics British Columbia Cancer Agency Victoria British Columbia Canada

**Keywords:** intensity‐modulated arc therapy, volumetric modulated arc therapy, RapidArc, treatment planning

## Abstract

RapidArc is a novel treatment planning and delivery system that has recently been made available for clinical use. Included within the Eclipse treatment planning system are a number of different optimization strategies that can be employed to improve the quality of the final treatment plan. The purpose of this study is to systematically assess three categories of strategies for four phantoms, and then apply proven strategies to clinical head and neck cases. Four phantoms were created within Eclipse with varying shapes and locations for the planning target volumes and organs at risk. A baseline optimization consisting of a single 359.8° arc with collimator at 45° was applied to all phantoms. Three categories of strategies were assessed and compared to the baseline strategy. They include changing the initialization parameters, increasing the total number of control points, and increasing the total optimization time. Optimization log files were extracted from the treatment planning system along with final dose‐volume histograms for plan assessment. Treatment plans were also generated for four head and neck patients to determine whether the results for phantom plans can be extended to clinical plans. The strategies that resulted in a significant difference from baseline were: changing the maximum leaf speed prior to optimization (p<0.05), increasing the total number of segments by adding an arc (p<0.05), and increasing the total optimization time by either continuing the optimization (p<0.01) or adding time to the optimization by pausing the optimization (p<0.01). The reductions in objective function values correlated with improvements in the dose‐volume histogram (DVH). The addition of arcs and pausing strategies were applied to head and neck cancer cases, which demonstrated similar benefits with respect to the final objective function value and DVH. Analysis of the optimization log files is a useful way to intercompare treatment plans that have the same dose‐volume objectives and importance values. The results for clinical head and neck plans were consistent with phantom plans.

PACS number: 87.55.x, 87.55.D, 87.55.de 87.55.dk

## I. INTRODUCTION

Intensity‐modulated arc therapy (IMAT) was initially proposed as an alternative to tomotherapy in 1995.^(1)^ Since then, a variety of different IMAT algorithms have been proposed that can be broadly categorized into two groups based on how the optimization problem is formulated. In one group are the studies that in one step optimize the multi‐leaf collimator (MLC) shapes and/or dose rate of each control point of an arc.^(^ ^2^ ^–^ ^5^ ^)^ In the other group are the studies which have formulated the optimization problem over two steps. In the first step, the fluence patterns are optimized for each control point of an arc; in the second step, the optimized fluence patterns are sequenced to find deliverable MLC patterns that can be delivered while respecting leaf motion constraints.^(^ ^1^ ^,^ ^6^ ^–^ ^8^ ^)^ Each of the groups are subject to their own unique challenges. One‐step plans are subject to being stuck in local minima^(9)^, while two‐step plans need to be leaf sequenced which can lead to degradation in plan quality.^(10)^

Volumetric‐modulated arc therapy (VMAT) is an arc therapy technique that is optimized in one step with a progressive sampling algorithm. This is a technique whereby the optimization begins with very coarse sampling of arc control points and then, as the optimization progresses, additional arc control points are added such that the final treatment plan has control points which are sampled at gantry positions approximately every 2°.^(4)^ The two main advantages to progressive sampling are that it reduces the optimization time and that it circumvents the highly restrictive leaf motion constraints early in the optimization by exploring an arc that is coarsely sampled and, therefore, allows for large leaf movements between successive coarse samples. Varian (Varian Medical Systems, Palo Alto, CA, USA) have implemented RapidArc as their version of VMAT.

RapidArc optimization is implemented similarly to Otto's VMAT algorithm^(4)^ with some differences. One difference is that VMAT adds arc control points one at a time during optimization, whereas RapidArc adds additional arc control points in groups which are called multi‐resolution (MR) levels. For example, for a 359.8° arc optimization within RapidArc, the number of arc control points is 10 during the first level of the multi‐resolution optimization (MR1), 21 during MR2, 43 during MR3, 87 during MR4, and 175 during MR5. Because control points are optimized for the middle of arc segments, two bounding control points must be added to define the start and stop positions for the arc, resulting in 177 control points. A second difference is that VMAT and RapidArc examine different numbers of changes per iteration. In VMAT, only one stochastic change of either MLC position or dose rate per iteration is allowed whereas, with RapidArc, there are seven random changes to either dose rate or MLC positions per iteration.^(11)^

In order to create plans for delivery, there are three interrelated machine parameters that are allowed to vary: the MLC leaf speed, the gantry speed, and the dose rate. The MLC leaf speed is kept within a prespecified maximum tolerance of 2.5 cm/s during the optimization. The gantry speed is then maximized at 4.8°/s unless the required MU per degree exceeds the maximum dose rate of 600 MU/min, in which case the gantry slows down to accommodate the required MU per degree.^(11)^

A challenging aspect of evaluating a commercial treatment planning system such as RapidArc is that many of the internal components are not fully described to their users. A systematic approach that analyzes both the inputs (optimization strategies and parameters) and outputs (final objective function value and dose volume histograms) can help users understand the behavior of such systems. The goal of this study is to assess various RapidArc planning strategies for a set of water equivalent phantoms and ultimately identify RapidArc strategies that consistently improve plan quality. Some of the strategies that have been proven to improve plan quality in phantoms will then be applied to clinical head and neck cases, and compared with a baseline planning strategy.

## II. MATERIALS AND METHODS

### A. Assessment of planning strategies for phantoms

#### A.1 Virtual phantoms for treatment planning systems

**Figure 1:**
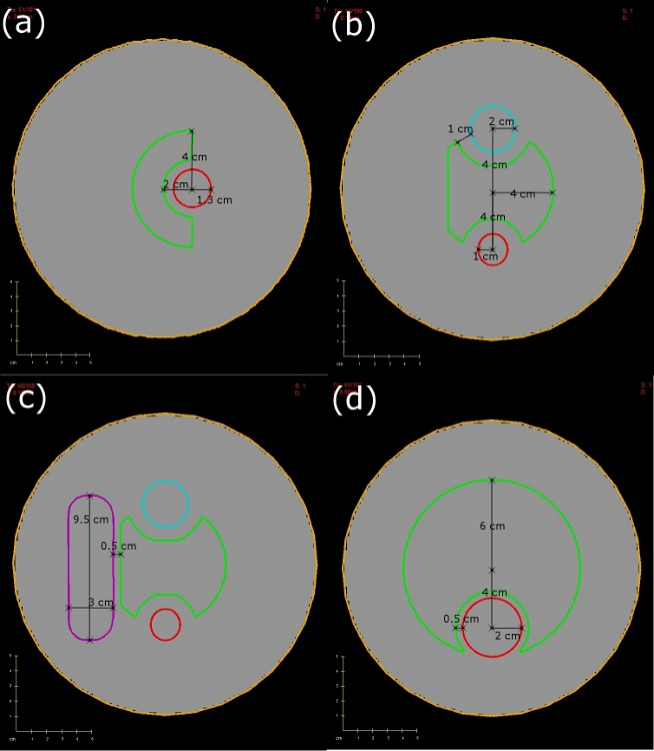
Axial slices of all phantoms with PTV and OARs shown: (a) Phantom 1 with PTV (green) and OAR1 (red); (b) Phantom 2 with PTV (green) with OAR1 (red) and OAR2 (blue); (c) Phantom 3 with PTV (green) with OAR1 (red), OAR2 (blue) and OAR3 (purple); (d) Phantom 4 (Brahme phantom) with PTV (green) and OAR (red).

Four virtual phantoms were created within the Eclipse planning environment (Eclipse 8.6.10, Varian Medical Systems, Palo Alto, CA). All virtual phantoms had a water equivalent density and had a 10.0 cm radius and a length of 25.25 cm. The contours of the planning target volume (PTV) and the organs at risk (OAR) were different on each phantom. The contours were two‐dimensional contours extended in the superior‐inferior dimensions, from the central slice, with the basic geometry as seen on an axial view (shown in Fig. 1). For Phantom 1, the superior‐inferior lengths of PTV and OAR were 8.5 cm and 6 cm, respectively. For Phantoms 2 and 3, PTV lengths were 8 cm and OAR1, OAR2, and OAR3 lengths were 5.25 cm. Finally, for Phantom 4, the PTV and OAR lengths were 8 cm and 7.5 cm, respectively, resulting in geometries similar to those used by Bortfeld and Webb.^(12)^

#### A.2 RapidArc optimization strategies for phantom cases

Plans were generated within RapidArc (Eclipse 8.6 prerelease version) with dose matrices that cover the entire external contour of the phantom with a voxel resolution of $0.25\times 0.25\times 0.25\;{\text{cm}}^{3}$. The prescription dose was 60 Gy in 30 fractions. The dose‐volume objectives and priorities used in the optimization are included in Table 1, and were kept the same for all the planning strategies investigated in this report. A baseline treatment plan was created that uses the standard parameters and is compared to strategies that alter the initialization parameters, increase the total number of segments, or increase the total time during optimization. A summary of the details and rationale for each of the strategies is included in Table 2, while additional details are included in Sections A.2.1.–A.2.3.

**Table 1:** All dose volume criteria used during optimization of Phantoms 1–4. All priority values were between 50 and 150 and were consistent for each phantom.

	*PTV*		*OAR1*			*OAR2*			*OAR3*		
	*Min Dose (Gy)*	*Max Dose (Gy)*	*DVH Vol (%)*	*DVH Dose (Gy)*	*Max Dose (Gy)*	*DVH Vol (%)*	*DVH Dose (GY)*	*Max Dose (Gy)*	*DVH Vol (%)*	*DVH Dose (Gy)*	*Max Dose (GY)*
P1	60.0	65.0	50.0	13.3	20.0						
P2	60.0	65.0	50.0	10.0	20.0	50.0	10.0	20.0			
P3	60.0	65.0	50.0	10.0	20.0	50.0	10.0	20.0	50.0	15.0	30.0
P4	60.0	65.0	50.0	15.0	30.0						

**Table 2:** A detailed summary of the optimization strategies, specific details regarding parameter changes, and rationale for making changes to that parameter are listed.

*Strategy*	*Details*	*Rationale*
Baseline	Collimator at 45°, 359.8° arc range, optimization run once through	Used as baseline comparison
Initialization – MLC shape	Use “fit and shield” to set MLC to PTV outline or PTV shielding OAR at each control point	Algorithm should produce similar solutions independent of initial conditions
Initialization – Collimator Rotation	Collimator at 0°, 15°, 30°, 90° and “Field Geometry Option” used	Collimator angles other than 45° might be better
Initialization – Increase maximum leaf speed	Increase maximum leaf speed from 2.5 cm/s to 3.0, 3.5 and 4.0 cm/s	Increasing leaf speed will allow for leaves to move further for the same control points
Increase Time for Opt – Continue Optimization	Run optimization once, continue optimization and return to MR2	Optimization time is increased so stochastic optimization process can be improved
Increase Time for Opt – Pause Optimization	Pause optimization for 7.5 or 15 minutes distributing the time evenly, incrementally, 1/3 MR4 and 2/3 MR5 or all in MR5	Increasing time will improve the plan quality due to stochastic component to optimization
Increase Control Points – Break up arcs into sub‐arcs	Two 180° arcs would produce 194 control points and three 120° arcs would produce 291 control points	Increasing number of control points should increase plan quality
Increase Control Points – Add additional arc	Add another arc with MLC leaves parallel to (collimator at 45°) or orthogonal to (collimator at 135°) leaves from first arc	Additional arc increases the total number of control points and should increase plan quality

##### A.2.1 Baseline optimization using standard conditions

The “baseline” optimization consisted of a single 359.8° arc with the Millennium 120 leaf MLC rotated to 45°. Within RapidArc, the “Arc Optimization” progressed through the optimization once to completion without interruptions and the “Normal Tissue Objective” (a parameter that limits dose as a function of distance from the PTV outer border) was not used because it would affect the objective function values. (Note that the quoted names are functions within RapidArc.)

##### A.2.2 Strategies with alternative initialization parameters

Three initialization strategies were assessed and compared to the baseline. They included changing the initial shapes of the MLC, changing the collimator angle, and increasing the maximum MLC speed.

The initial MLC apertures prior to optimization may be important if the optimization algorithm is not capable of overcoming local minima. To test the effect of MLC initialization, the initial MLC shapes were set to encompass either the PTV alone or the PTV but shielding the OARs using the “fit and shield” option. This was done to determine whether any MLC initialization strategy is preferred or if the final plan is independent of the MLC initialization.

The collimator was rotated to angles of either 0°, 15°, 30° or 90° for four plans, while a fifth plan used the “Field Geometry Optimization” option which chooses an isocenter, couch and collimator placement prior to optimization.

The effect of maximum leaf speed on plan quality was tested by changing it from the nominal value of 2.5 cm/s to 3.0 cm/s, 3.5 cm/s, and 4.0 cm/s. The mechanical limit as specified by Varian is 3.0 cm/s for the Millennium 120 MLC. It is important to note that plans that exceed this limit may not be clinically deliverable.

##### A.2.3 Strategies that increase the total number of control points

Two strategies were used to increase the total number of control points. The first is to divide arcs into sub‐arcs and the second is to add an additional 359.8° arc.

When the arc was defined as either two 180° sub‐arcs or three 120° sub‐arcs, the total number of control points increased from 177 for 1 arc to 194 or 291 for the 2 or 3 sub‐arc plans.

Adding a second 359.8° arc doubles the total number of control points from 177 to 354. This was done with either the collimator set to either 45° (leaf motion parallel to the first arc) or set to 135° (leaf motion orthogonal to the first arc).

##### A.2.4 Strategies that increase the total optimization time

Strategies that increase the total optimization time were considered desirable because optimization in RapidArc normally terminates after a fixed number of steps rather than at some tolerance level of the objective function (OF), resulting in plans that can be further optimized. Two strategies that increase the total optimization time were: continuing the optimization after one optimization has completed, or pausing the optimization at specific multi‐resolution levels for an additional 7.5 minutes or 15 minutes.

There is an option within RapidArc to continue the previous optimization. When selected, the optimization continues on the highest multi‐resolution level (MR5) of the multi‐resolution optimization. In this investigation, plans were moved back to MR2 in order to give the algorithm more time to optimize with a coarser set of control point samplings. This may be advantageous because when moved back to MR2, the intermediate control points have already been considered in the previous optimization.

**Figure 2:**
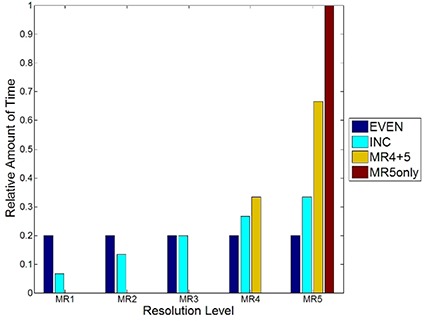
A plot of the distribution of how the relative amount of time is distributed for each of the pausing strategies employed in this study.

There is also a capability within RapidArc to pause the optimization for a longer period of time on any desired resolution level. The effect on the plan quality of such a strategy was tested by allocating either an extra 7.5 minutes or 15 minutes to the optimization. Since the most efficient way to distribute this additional time is not known, four distinct strategies were assessed. The first strategy (EVEN) involved distributing the additional time evenly throughout the levels, which allowed for an additional 1.5 or 3 minutes per level. The second strategy (INC) involved pausing the optimization on each level but distributing more time to the latter levels according to the following incremental schedules: 0.5, 1, 1.5, 2, 2.5 minutes or 1,2,3,4,5 minutes for MR levels 1–5. The third strategy (MR4+5) distributed the time such that 1/3 of the pause time is within MR4 and 2/3 of the time is in MR5. Finally, the fourth strategy (MR5only) involved pausing the optimization within MR5 only. Figure 2 demonstrates how the time is distributed for the four pausing strategies.

#### A.3 Evaluation of planning strategies

All planning strategies were evaluated based on their final objective function value reported in the optimization log file created by the Eclipse planning system at the completion of optimization. For each phantom, the planning strategies were compared to the baseline strategy by determining the relative objective function value (ROFV) as shown in Eq. 1:

(1) $$\text{ROF}V_{x}=\frac{OFV_{X}}{OFV_{\text{bas}\text{eli}ne}}$$

where ${\text{OFV}}_{x}$ is the objective function value for the strategy that is being assessed and ${\text{OFV}}_{\text{baseline}}$ is the objective function value for the baseline plan as described in Section A.2.1. above. If the ROFV is more than 1, then the planning strategy being assessed is worse than baseline; if it is less than 1, then it is better than baseline. The average ROFV for all four virtual phantoms were compared for each strategy against baseline using paired Student's t‐tests to determine which strategies were significantly different.

The final dose distribution was exported from the planning system and imported into Computational Environment for Radiotherapy Research (CERR) version 3.0 for dose‐volume evaluation.^(13)^ The DVHs were analyzed to determine whether the decreases in the ROFV correlated with improvements in the DVHs.

### B. Apply effective strategies to clinical cases

Single arc RapidArc plans were generated for four head and neck cancer patients (Case 1: oropharynx with unilateral nodes, Case 2: larynx with bi‐lateral nodes, Case 3 and 4: oropharynx with bi‐lateral nodes) using the baseline strategy described in section A.2.1. These plans were compared to plans generated using the most effective planning strategy in phantoms. An additional arc was added with collimator parallel and pausing the optimization for either 7.5 or 15 minutes using an incremental pausing schedule. These strategies were chosen because adding control points by adding an arc and adding optimization time using an incremental pausing strategy were determined to be effective for the phantom cases. The prescription dose was 60 Gy in 25 fractions to the primary PTV60 and 50 Gy in 25 fractions to the lymph nodes PTV50. The organs at risk considered in this study were the spinal cord and parotid glands.

Evaluation of the clinical head and neck treatment plans were based on the final objective function value along with dose‐volume histogram analysis. Additional metrics that were assessed included the overall treatment planning time, estimated treatment delivery time, total MU, and number of optimization iterations.

## III. RESULTS

### A. Assessment of strategies for the phantom studies

**Figure 3:**
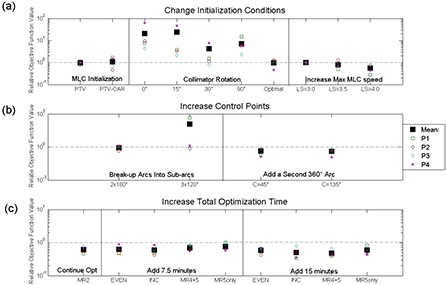
A plot of the relative objective function value for the strategies where: (a) the initialization conditions were changed; (b) the number of control points was increased; and (c) the total optimization time was increased. The markers: a large solid box (the mean), an open box (Phantom 1 (P1)), a circle (P2), a diamond (P3), an asterisk (P4). A baseline plan would correspond to a ROFV of 1 and is represented by a dotted gray line.

Figure 3 summarizes the relative objective function values for changing the initialization, increasing the number of control points, and increasing the optimization time. For the initialization strategies, only increasing the maximum leaf speed to 4.0 cm/s was significantly different than baseline ${(\text{ROFV}}_{\text{LS}=4}=0.549\pm 0.235,p<0.05)$. For the strategies that increase the total number of control points, adding an arc with a collimator at either $45{}^{\circ}\;{(\text{ROFV}}_{\text{Add Arc, C}45}=0.434\pm 0.317,p<0.05)$ or $135{}^{\circ}\;{(\text{ROFV}}_{\text{Add Arc, C}45\&135}=0.425\pm 0.230,p<0.05)$ resulted in significant differences from baseline. For the strategies that increase the total optimization time, continuing the optimization from level 2 ${(\text{ROFV}}_{\text{ContMR}2}=0.609\pm 0.133,p<0.05)$ and pausing the optimization for 7.5 minutes and 15 minutes resulted in significantly reduced objective function as compared to baseline.

When employing the pausing strategies, the distribution of time among the resolution levels was important. Adding an additional 7.5 minutes was significantly different for even time among resolution levels ${(\text{ROFV}}_{7.5\min,\text{EVEN}}=0.626\pm 0.191,p<0.05)$, incremental amounts of time added to all resolution levels ${(\text{ROFV}}_{7.5\min,\text{INC}}=0.595\pm 0.186,p<0.05)$, levels MR4 and MR5 ${(\text{ROFV}}_{7.5\min,\text{MR}4+5}=0.698\pm 0.146,p<0.05)$ but not for MR5. For the addition of 15 minutes, the evenly distributed ${(\text{ROFV}}_{15\min,\text{EVEN}}=0.588\pm 0.115,p<0.01)$, incrementally distributed ${(\text{ROFV}}_{15\min,\text{INC}}=0.498\pm 0.216,p<0.05)$, distributed among MR4&5 ${(\text{ROFV}}_{15\min,\text{MR}4+5}=0.482\pm 0.121,p<0.01)$ and among MR5 ${(\text{ROFV}}_{15\min,\text{MR}5\text{ONLY}}=0.590\pm 0.193,p<0.05)$ strategies were all significantly different from the baseline plan. Although all strategies significantly reduced the ROFV, only the incremental pausing strategy was applied to the clinical cases because it was effective at lowering the ROFV for both 7.5 minutes and 15 minutes.

**Figure 4:**
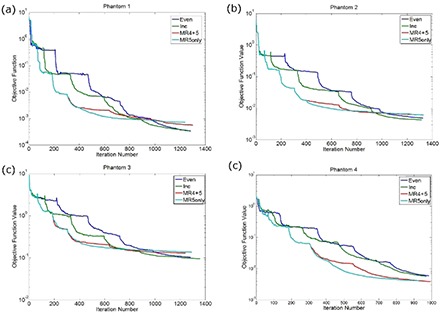
A plot demonstrating the effect of adding an additional 7.5 minutes by pausing the optimization with varying amounts of time in each resolution level for (a) Phantom 1, (b) Phantom 2, (c) Phantom 3, and (d) Phantom 4.

The OF value, as a function of the iteration number for each phantom, is shown in Fig. 4 for the 7.5 and 15 minute pausing strategies. The step‐patterns in Fig. 4 correspond to the different multi‐resolution levels during the optimization.

### B. Relationship between objective function and DVH for phantom cases

**Figure 5:**
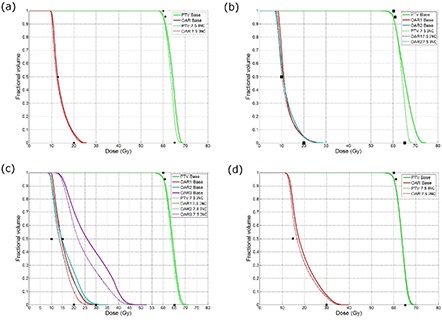
Plots of dose‐volume histograms for plans run with the baseline strategy (solid) as compared to plans generated with the incremental pausing strategy where the total pausing time is 7.5 minutes for (a) Phantom 1, (b) Phantom 2, (c) Phantom 3 and (d) Phantom 4. The black points represent the desired dose‐volume histogram points. The absolute objective function values for each plan and strategy are as follows; OFVBASE=0.00071 vs. OFV7.5LIN=0.00034 for Phantom 1; OFVBASE=0.010 vs. OFV7.5LIN=0.0042 for Phantom 2; OFVBASE=0.15 vs. OFV7.5LIN=0.095 for Phantom 3; OFVBASE=0.0067 vs. OFV7.5LIN=0.0057 for Phantom 4.

**Figure 6:**
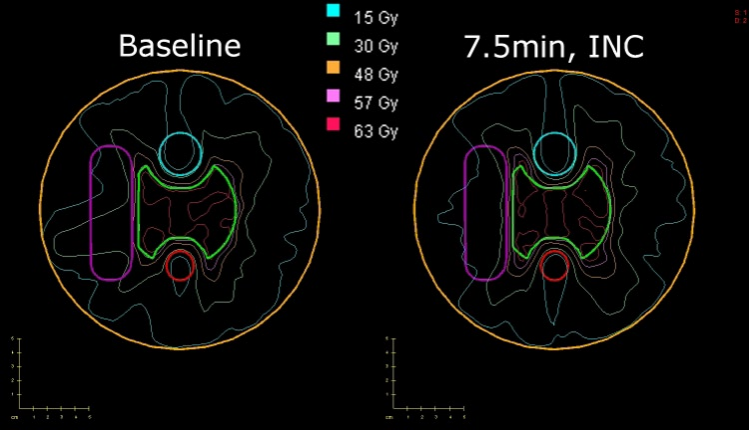
A plot with PTV and OAR contours with isodose lines at 105% (63 Gy), 95% (57 Gy), 80% (48 Gy), 50% (30 Gy), and 25% (15 Gy) for 1 run (left) and a 7.5‐minute pausing strategy (right) for Phantom 3.

Dose distributions and dose‐volume histograms for baseline plans are compared with the strategy where the time spent per level increments with the resolution level number. All dose volume histograms along with the desired DVH points that were used during optimization are included in Fig. 5, along with the final OF value in the figure caption. Figure 6 shows axial views of isodose lines for 25%, 50%, 80%, 95% and 105% of the prescription dose for Phantom 3.

### C. Application of baseline, add arc and add time to clinical cases

**Figure 7:**
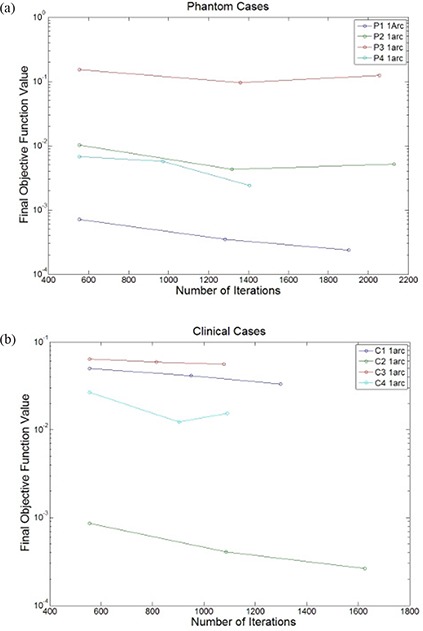
A plot of the final objective function with the total number of iterations for (a) the phantom cases (P1–P4) and (b) clinical cases (C1–C4) using a strategy that pauses the optimization at each resolution level with incremental amounts of time added to later levels.

**Figure 8:**
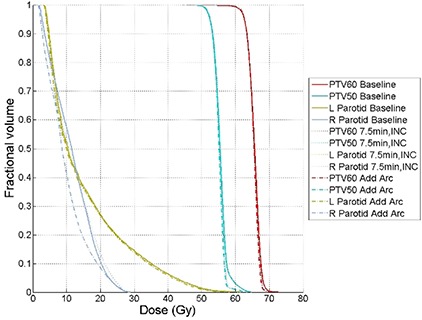
Dose volume histograms for the treatment plans that included single arc baseline plan (Baseline, solid), single arc plan with incremental 15 minute pausing schedule (7.5 min, INC, dotted), dual arc plan (Add Arc, dash dotted) for clinical case 1. All structures are listed in the legend. The objective function values for this plan are: 0.050 for Baseline, 0.033 for 7.5 min, INC and 0.037 for Add Arc.

A summary of the final OF value, total number of monitor units, optimization time, time for final dose calculation, estimated treatment time, and total number of iterations are reported in Table 3 for all four head and neck cases. A figure showing the final OF value as a function of the total number of iterations is shown for one arc plan to the phantom studies in Fig. 7(a) and to the single arc plans for the clinical cases in Fig. 7(b). For one of the clinical cases, Fig. 8 shows the dose volume histograms for the baseline optimization run, an optimization run where the optimization is paused for 15 minutes according to an incremental schedule and the addition of another arc.

**Table 3:** A table summarizing the results from the assessment of the clinical cases, the final objective function, total number of monitor units, the optimization time, time for final dose calculation, and the total number of iterations are reported. All times have units of minutes. The estimated treatment time is 1.25 minutes for all single arc plans and 2.50 minutes for every dual arc plan.

*Case*	*No. of Arcs*	*Strategy*	*Final OF value*	*Total MU*	*Opt Time*	*Dose Calc Time*	*No. of Iterations*
1	1	Baseline	0.050	616	15.6	12.5	555
		7.5 min INC	0.041	597	23.7	12.5	838
		15 min INC	0.033	631	31.6	12.5	1127
	2	Add arc	0.037	633	24.4	26	839
2	1	Baseline	0.00086	575	11.6	10	555
		7.5 min INC	0.00041	623	18.6	10	952
		15 min INC	0.00026	541	28.9	10	1621
	2	Add arc	0.000037	592	17.6	20	839
3	1	Baseline	0.064	627	22.0	12	555
		7.5 min INC	0.059	627	29.5	12	816
		15 min INC	0.056	606	26.4	12	938
	2	Add arc	0.045	667	34.6	25	839
4	1	Baseline	0.027	423	20.0	17.5	555
		7.5 min INC	0.012	477	28.3	17.5	756
		15 min INC	0.015	473	34.0	17.5	1091
	2	Add arc	0.0048	556	31.3	35	839

## IV. DISCUSSION

The only strategy that produced significantly different final OF values from baseline for alternative initialization parameters was increasing the maximum leaf speed to 4.0 cm/s. Although this leaf speed is outside of the limits recommended by the vendor for this linear accelerator, this plan may be realized (with increased time) by reducing the maximum dose rate, the only rate parameter which is configurable by the user. The best strategies for increasing the number of control points consisted of adding a 359.8° arc with the collimator rotated to either 45° or 135°. The best strategies that added additional time to the optimization included: continuing the optimization and returning to MR2; pausing the optimization for 7.5 or 15 minutes while distributing the time evenly throughout the resolution levels, incrementing the time to each resolution levels (in resolution levels 4 and 5 only). No significant improvement was seen if time was added entirely in resolution level 5 for pausing the optimization for 7.5 minutes.

There were no strategies that resulted in final OF values significantly higher than the values from the baseline strategy. The strategies that were not significantly different than baseline fell mostly within the initialization parameters category and included collimator configurations other than 45°, the field geometry optimization option, or changing the MLC initialization. For the collimator configurations other than 45°, collimator settings of 0°, 15° and 90° all produced ROFV values greater than 5. However, none of these were significantly different than baseline, suggesting for these geometries that a collimator angle of 45° is near optimal. The strategies that broke up the arc into sub‐arcs produced more control points. However, the values were not significantly lower, perhaps due to the MLC restrictions that are present for shorter arcs due to densely packed control points. Our results indicate that the optimization is not dependent on the MLC initialization prior to optimization, indicating that the algorithm is not being limited by the initial choice of the MLC shape. Our results also confirm that the optimal collimator angle is around 45°. This result is quite important as the OF values at 0°, 15° and 90° produced plans that were approximately an order of magnitude higher than the baseline plan (as shown in Fig. 3). The only initialization strategy that could improve the final OF value was increasing the maximum leaf speed to speeds that violate the manufacturer's recommendations. In addition, adding another arc and adding time to the optimization were both beneficial; however, these strategies lengthen both the optimization time and/or treatment time.

The “MU objective” was an additional initialization parameter that was investigated (not described in the methods section). The “MU Objective” is used to control the number of MUs delivered and is used with an upper and lower bound and a “strength” parameter which controls the convergence of the “MU Objective”. Using this objective with various upper bound, lower bounds, and strength parameters resulted in insignificant differences as compared to baseline. However, it was useful in limiting the total number of MUs.

There is a strong relationship between the magnitude of the final OF value and the quality of the dose volume histogram, as is shown in Fig. 5. In Figs. 5(a) and 5(b), the PTV dose uniformity is improved and, in Figs. 5(c) and 5(d), the dose to the OAR is lowered when the 7.5 minute linear pausing strategy is applied.

The clinical cases demonstrated similar trends in reducing the final OF value as compared to the phantom cases as is shown in Fig. 7(b). For cases 1 and 3, adding an additional arc does not improve the plan any more than pausing the plan for an additional 15 minutes, as demonstrated by the final OF value shown in Table 3. As is shown in Fig. 7(b), the benefits of adding an additional arc are more consequential for cases 2 and 4, where approximately an order of magnitude decrease in the OF value is observed. Although the clinical cases demonstrate a considerable reduction of the final OF value, it is not as apparent when looking at the DVHs because there are often multiple PTVs and OARs in clinical head and neck cases, whereas the phantom cases had a single PTV and between 1 to 3 OARs.

The results presented in this study are only applicable if the dose‐volume objectives and related priorities are kept constant, in which case the comparison will be valid. If different dose‐volume objectives and priorities are used for an intercomparison, then the objective function values can not be compared, but the dose‐volume histograms and other traditional dose based metrics are still valid.

Although we applied a pausing strategy based on pausing each resolution level with an incrementing amount of time, other strategies may have provided better results. However, the user must understand that when creating treatment plans for RapidArc, there is a trade‐off between plan quality and the amount of time spent planning, and this balance needs to be assessed by each user.

Increasing the mechanical limitations of the hardware for the linear accelerator can increase the quality of the resulting treatment plans. As is demonstrated in these results, increasing the maximum leaf travel speed above the recommended 2.5 cm/s can result in increased plan quality. However, delivering above 3.0 cm/s is above the technical specification set by Varian for their Millennium multi‐leaf collimator and attempting to deliver above this limit will not be allowed by the delivery system. Furthermore, it has been shown that there is a relationship between the leaf speed and the inaccuracy of the leaf positions for arc therapy such that at higher leaf speeds, the magnitude of positional errors increases.^(^ ^14^ ^,^ ^15^ ^)^

The results presented in this work are applicable to the RapidArc version within Eclipse version 8.6 only. As algorithmic advances in RapidArc optimization (and other arc optimization techniques) become commercially available, the results of this study may no longer be applicable. However, the methods from this study can be obtained and used to better understand and characterize these newer algorithms.

Future work will involve determining whether the results from this study can be extended to additional clinical geometries.

## V. CONCLUSIONS

The goal of this paper was to determine strategies within RapidArc that would be effective at reducing the final OF value and, thereby, improving final plan quality. Significant differences as compared to a baseline strategy included increasing the maximum leaf speed to 4.0 cm/s, doubling the number of control points by adding another arc with leaf motion either parallel or perpendicular to the initial arc, and increasing the optimization time by continuing the optimization at MR2 or adding time to the different resolution levels during optimization. The magnitudes of the decreases in the significantly different strategies were between 40%–55% the final OF values produced by the baseline strategy. The reductions in final OF values were correlated with improvements in the dose‐volume histogram for the phantoms. The addition of arcs and pausing strategies were applied to four head and neck cancer cases, which demonstrated similar improvements in plan quality.

## References

[1] Yu CX . Intensity‐modulated arc therapy with dynamic multileaf collimation: an alternative to tomotherapy. Phys Med Biol. 1995;40(9):1435–49. 8532757 10.1088/0031-9155/40/9/004

[2] Earl MA , Shepard DM , Naqvi S , Li XA , Yu CX . Inverse planning for intensity‐modulated arc therapy using direct aperture optimization. Phys Med Biol. 2003;48(8):1075–89. 12741503 10.1088/0031-9155/48/8/309

[3] Ulrich S , Nill S , Oelfke U . Development of an optimization concept for arc‐modulated cone beam therapy. Phys Med Biol. 2007;52(14):4099–119. 17664597 10.1088/0031-9155/52/14/006

[4] Otto K . Volumetric modulated arc therapy: IMRT in a single gantry arc. Med Phys. 2008;35(1):310–17. 18293586 10.1118/1.2818738

[5] Zygmanski P , Högele W , Cormack R , Chin L , Löschel R . A volumetric‐modulated arc therapy using sub‐conformal dynamic arc with a monotonic dynamic multileaf collimator modulation. Phys Med Biol. 2008;53(22):6395–417. 18941279 10.1088/0031-9155/53/22/009

[6] Gladwish A , Oliver M , Craig J , et al. Segmentation and leaf sequencing for intensity modulated arc therapy. Med Phys. 2007;34(5):1779–88. 17555259 10.1118/1.2724064

[7] Luan S , Wang C , Cao D , Chen DZ , Shepard DM , Yu CX . Leaf‐sequencing for intensity‐modulated arc therapy using graph algorithms. Med Phys. 2008;35(1):61–69. 18293562 10.1118/1.2818731

[8] Wang C , Luan S , Tang G , Chen DZ , Earl MA , Yu CX . Arc‐modulated radiation therapy (AMRT): a single‐arc form of intensity‐modulated arc therapy. Phys Med Biol. 2008;53(22):6291–303. 18936519 10.1088/0031-9155/53/22/002

[9] Oefke U , Nill S , Wilkens JJ . Physical Optimization. In: Bortfeld T, Schmidt‐Ulrich R, DeNeve W, Wazer D, editors. Image‐guided IMRT. Belin (DE): Springer Verlag; 2007.

[10] Ludlum E , Xia P . Comparison of IMRT planning with two‐step and one‐step optimization: a way to simplify IMRT. Phys Med Biol. 2008;53(3):807–21. 18199916 10.1088/0031-9155/53/3/018

[11] Cozzi L , Dinshaw KA , Shrivastava SK , et al. A treatment planning study comparing volumetric arc modulation with RapidArc and fixed field IMRT for cervix uteri radiotherapy. Radiother Oncol. 2008;89(2);180–91. 18692929 10.1016/j.radonc.2008.06.013

[12] Bortfeld T , Webb S . Single‐Arc IMRT? Phys Med Biol. 2009;54(1):N9–N20. 19075362 10.1088/0031-9155/54/1/N02

[13] Deasy JO , Blanco AI , Clark VH . CERR: a computational environment for radiotherapy research. Med. Phys. 2003;30(5):979–85. 12773007 10.1118/1.1568978

[14] Ramsey CR , Spencer KM , Alhakeem R , Oliver AL . Leaf position error during conformal dynamic arc and intensity modulated arc treatments. Med Phys. 2001;28(1):67–72. 11213924 10.1118/1.1333410

[15] Ramsey CR , Usynin A , Chase D . Multileaf collimator performance and quality assurance for volume modulated arc therapy [Abstract]. Med Phys. 2008:35(6):2909.

